# Corticosteroid response and biopsy decisions in tumefactive demyelination and primary central nervous system lymphoma

**DOI:** 10.3389/fimmu.2026.1988759

**Published:** 2026-10-14

**Authors:** Xu Wang, Shougang Guo

**Affiliations:** Department of Neurology, Shandong Provincial Hospital Affiliated to Shandong First Medical University, Jinan, Shandong, China

**Keywords:** brain biopsy, corticosteroids, magnetic resonance imaging, neuroimmunology, primary central nervous system lymphoma, targetability, tumefactive demyelination

## Abstract

Distinguishing primary central nervous system lymphoma (PCNSL) from tumefactive demyelination becomes especially difficult after corticosteroid treatment. Both conditions may improve clinically and radiologically, while corticosteroids can reduce lymphoma cellularity, alter enhancement, and change the tissue available for sampling. The biopsy literature reflects this time dependence. Meta-analysis shows a higher average risk of nondiagnostic tissue after prebiopsy exposure, yet contemporary cohorts report high diagnostic yields in selected patients when enhancement persists and current imaging guides prompt sampling. We organize the evidence around four time-linked clinical questions. Before corticosteroids (T0), clinicians establish the competing probabilities and preserve baseline anatomy. After treatment (T1), they measure the anatomical and clinical transformation. Before biopsy (T2), current imaging should determine whether a representative target remains visible, safely accessible, and sufficiently sampleable. A persistent useful target supports prompt, imaging-updated biopsy; marked regression calls for planned short-interval imaging to recover a representative target. Cerebrospinal fluid biomarkers can modify probability, although single-marker negativity is weak and composite results remain prevalence- and platform-dependent. After biopsy (T3), pathology must fit the sampled site and subsequent course. A major verified inconsistency should reopen imaging–pathology review and prompt repeat biopsy when a useful target is available. This approach treats corticosteroid response as a measurable change in diagnostic substrate and links each decision to the evidence available at that time.

## 1 Introduction

A patient with a mass-like brain lesion improves quickly after corticosteroids, and the enhancing lesion shrinks. When tumefactive demyelination and primary central nervous system lymphoma (PCNSL) are the leading possibilities, that response leaves the central diagnosis unresolved. Tumefactive demyelinating lesions (TDLs) often respond, and PCNSL can also regress because lymphoma cells are glucocorticoid-sensitive (1–3). Treatment may relieve symptoms while reducing enhancement, depleting neoplastic B cells, and removing the most informative tissue from a later biopsy. Current PCNSL guidance recommends avoiding corticosteroids before biopsy when clinically possible (4). Mass effect, neurological deterioration, or treatment before specialist referral nevertheless makes prior exposure common in practice.

After exposure, the evidence appears to point in opposite directions. A systematic review and meta-analysis found a higher risk of nondiagnostic tissue after prebiopsy corticosteroids, particularly with stereotactic biopsy (3). In a four-center cohort that required persistent enhancement, diagnostic surgery succeeded in 68 of 70 exposed patients (5). A recent cohort using contrast MRI within 24–48 hours of stereotactic biopsy established the diagnosis in 41 of 43 exposed patients at the first procedure (6). The meta-analysis estimates average risk across heterogeneous eras, lesion states, and workflows. The modern cohorts mainly describe patients whose target remained visible and who proceeded to surgery. Together, they identify current lesion state and pathway selection as the bridge between population risk and an individual biopsy decision.

The practical question after corticosteroids is therefore, “What lesion is left now, and can it still provide a diagnosis?” We use biopsy targetability as shorthand for this preprocedural judgment. A useful target is likely to represent the process of concern, can be reached at acceptable risk, and can provide enough tissue for diagnosis. Specimen adequacy is judged after tissue has been obtained and remains a separate endpoint.

We focus on adults with a mass-like parenchymal CNS lesion in whom PCNSL and tumefactive inflammatory demyelination remain active competing diagnoses. Most post-corticosteroid biopsy-yield evidence comes from immunocompetent PCNSL cohorts. Immunosuppression broadens the differential diagnosis and changes the meaning of lesion regression. We organize the review around the T0–T3 framework: before corticosteroids (T0), after corticosteroids (T1), immediately before a possible biopsy (T2), and after pathology is available and follow-up begins (T3) (**Figure 1**).

**Figure 1:**
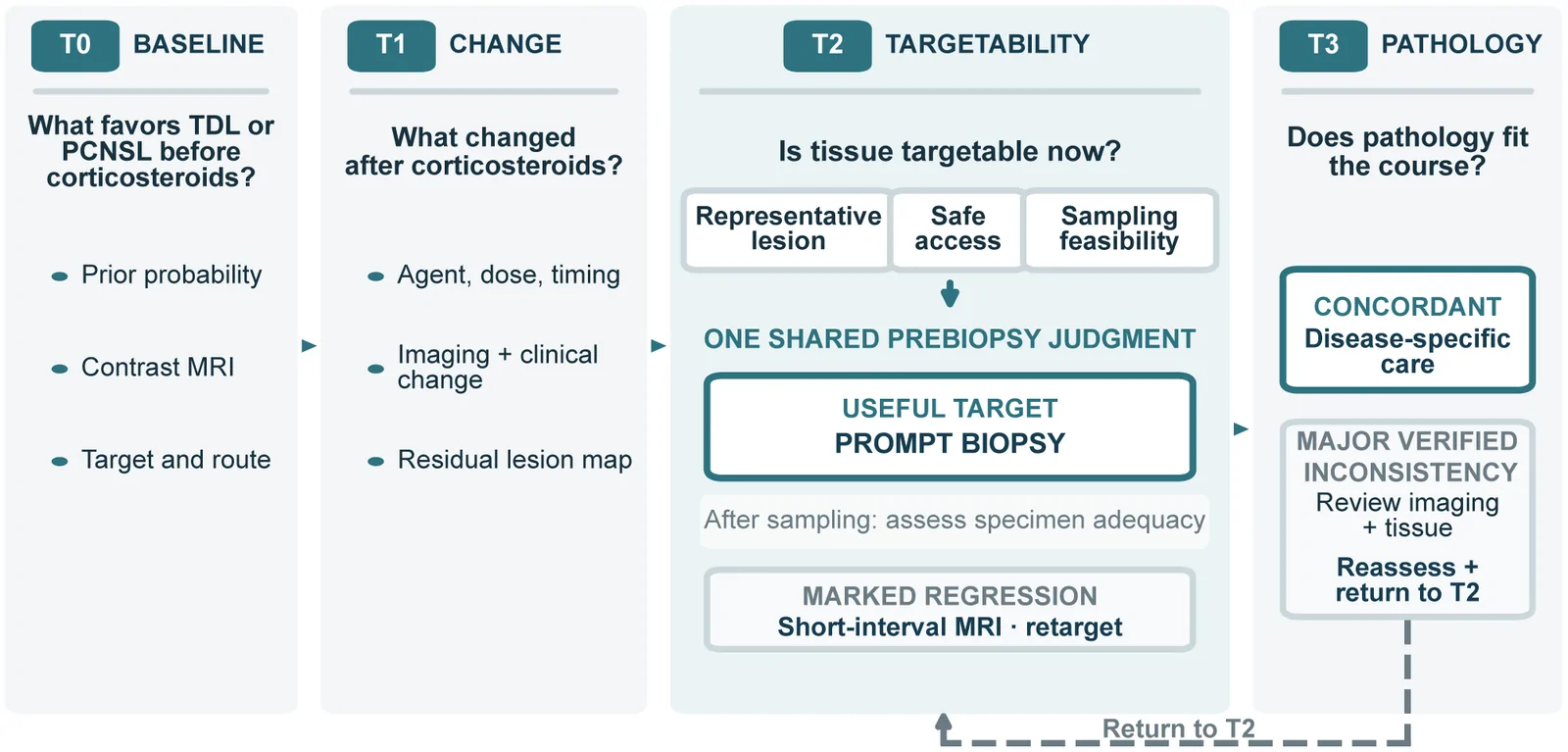
A time-dependent approach after corticosteroid exposure when tumefactive demyelination and primary central nervous system lymphoma remain competing diagnoses. T0 establishes prior probability and preserves baseline anatomy. T1 records the treatment-induced transformation. T2 integrates lesion selection (representative lesion), procedural feasibility (safe access), and sampling feasibility to determine whether a useful biopsy target remains. A useful target supports prompt biopsy; marked regression triggers planned short-interval MRI for retargeting. Specimen adequacy is assessed after tissue acquisition. T3 tests concordance among the sampled site, pathology, and subsequent course; a major verified inconsistency activates imaging–pathology review, retargeting, and repeat biopsy when feasible.

## 2 Methods

This focused narrative review was informed by targeted searches of PubMed/MEDLINE, Web of Science, Scopus, and Google Scholar through August 28, 2026. These searches were supplemented by Crossref, Europe PMC, publisher sources, and backward and forward citation tracking. Search concepts included tumefactive or pseudotumoral demyelination, PCNSL, sentinel lesions, corticosteroids, biopsy yield, neuropathology, diagnostic delay, repeat biopsy, cerebrospinal fluid (CSF) biomarkers, and longitudinal follow-up. We prioritized clinical guidance, systematic syntheses, direct TDL–PCNSL comparisons, neuropathological studies, biopsy cohorts with interpretable denominators, and recent evidence on CSF interleukin-10 (IL-10), *MYD88* L265P, and C-X-C motif chemokine ligand 13 (CXCL13). Evidence was selected purposively to address the T0–T3 questions. We did not perform PRISMA-style screening, formal risk-of-bias assessment, or a *de novo* meta-analysis.

## 3 T0. Before corticosteroids: how likely are TDL and PCNSL

Tumefactive demyelination is a clinicoradiological phenotype with several possible disease contexts. Large mass-like demyelinating lesions occur in multiple sclerosis, myelin oligodendrocyte glycoprotein antibody-associated disease (MOGAD), aquaporin-4-IgG-positive neuromyelitis optica spectrum disorder, acute disseminated encephalomyelitis (ADEM) and other inflammatory conditions (7, 8). In a comparative cohort of 108 patients, the clinical and imaging phenotype differed across MOGAD, aquaporin-4-IgG-positive disease, and multiple sclerosis (8). A scoping review of published TDL cases in adults aged ≥55 years found a median age of 62 years and a high biopsy rate (68%), in the setting of frequent initial concern for malignancy (9). Biopsy-confirmed series show that a first tumefactive event may evolve into a recognizable multiple-sclerosis course or remain isolated during follow-up (10). Histology can establish active demyelination; disease attribution still requires serology, lesion distribution, and longitudinal phenotype (11). A tumefactive appearance therefore raises the probability of inflammatory demyelination without identifying its cause.

An inflammatory interpretation becomes more persuasive when independent observations converge. A previous typical demyelinating attack, remote lesions with a characteristic distribution, disease-concordant MOG-IgG or aquaporin-4-IgG, supportive CSF oligoclonal bands, and a coherent clinical tempo strengthen an inflammatory interpretation. Older age, immunosuppression, and cognitive or behavioral change raise concern for PCNSL (4). Age alone has limited resolving power. An independently verified inflammatory disorder carries more weight than a previous label of “steroid-responsive demyelination”.

T0 should also identify diagnostic material outside the dominant parenchymal lesion. Ophthalmologic assessment, systemic imaging, and CSF examination may reveal a safer or independent route to lymphoma diagnosis when feasible (4). Their value lies in convergence across compartments. A positive CSF or ocular finding can raise PCNSL probability before corticosteroids alter the brain lesion, while negative staging leaves the parenchymal competition unresolved. The team should therefore complete feasible staging promptly without allowing it to delay an accessible, high-value tissue target.

Sentinel PCNSL illustrates the danger of closing the diagnosis on one reassuring observation. Regressing lesions and biopsy tissue showing inflammatory demyelination can precede a definitive diagnosis of PCNSL by months (12–14). Alderson et al. described four immunocompetent patients whose initial specimens showed demyelination, nonspecific inflammation, or normal tissue before B-cell PCNSL was diagnosed seven to eleven months later (12). A later synthesis combined two local cases with 14 published cases; repeat biopsy confirmed PCNSL in all 16 cases after a median interval of 10 months (range 3–60) (13). These selected reports document a clinically important diagnostic sequence without estimating the frequency of occult PCNSL among unselected TDLs. The term “sentinel lesion” refers to this retrospective clinical-pathological sequence.

MRI shifts probability through combinations of features. A meta-analysis of 19 studies including 476 patients with TDL reported pooled sensitivity of 89% and specificity of 94% for MRI-based differentiation from primary brain tumors; the comparator was not specifically PCNSL (15). Open or incomplete-ring enhancement occurred in 35% of TDLs. Its specificity can be helpful when present, but variable sensitivity limits its ability to exclude TDL when absent. In one pathological cohort, the MRI-enhancing region was hypoattenuating on unenhanced CT in 14 of 15 TDLs and 2 of 48 tumors; the tumor group contained lymphomas and gliomas (16). Peripheral restricted diffusion can occur at the active edge of demyelination (17). Very low minimum apparent diffusion coefficient values favor hypercellular PCNSL, whereas perfusion provides less consistent discrimination because both entities may show low relative cerebral blood volume (15, 18). Proton MR spectroscopy adds information but retains substantial overlap (19).

Multifeature interpretation is more informative than reliance on individual imaging signs. Chew et al. retrospectively compared 23 TDLs, 31 high-grade gliomas, and 12 CNS lymphomas. Two independent neuroradiologists reviewed presentation MRI while blinded to final diagnosis (20). Smaller size, limited mass effect, incomplete peripheral enhancement, absent central enhancement or central restricted diffusion, lack of cortical involvement, and remote white-matter lesions favored TDL. Only 6 of 23 TDLs were biopsy-confirmed. The remainder were classified through treatment response, lesion resolution within three months, and at least two years of follow-up. This approach provides clinically meaningful longitudinal validation but introduces incorporation bias because radiological improvement contributes to the definition of the TDL group. The small lymphoma subgroup, mixed tumor comparator, and incomplete image availability also limit the specificity of conclusions regarding PCNSL discrimination. The study supports pattern recognition as one component of biopsy decision-making.

Sun et al. reported one of the largest pathology-based comparisons, including 116 TDLs and 150 PCNSLs, with both groups described as pathologically confirmed (21). TDLs occurred at a younger age and were usually hypoattenuating on CT, whereas approximately 80% of PCNSLs were hyperattenuating. Reactive astrocytes, particularly Creutzfeldt–Peters cells, and macrophage-rich demyelination could mimic neoplasia, whereas steroid-treated or sentinel PCNSL could mimic inflammatory demyelination. The retrospective design and clinically selected cohort limit the generalizability of diagnostic modeling. The cohort nevertheless reinforces two practical points: CT attenuation can provide complementary information independent of MRI findings, and pathology should be interpreted in the context of corticosteroid exposure and the exact sampled site. A newer single-center cohort of 97 PCNSLs and 86 TDLs reported differences in age, MRI appearance, CD3/CD20 staining, and CSF IL-6 and IL-10 (22). Its proposed discriminators await external testing because TDL ascertainment criteria were heterogeneous and all patients came from a single center.

Longitudinal TDL cohorts show why classification should remain anchored to the underlying disease process. Across series, an initial tumefactive event later evolved into multiple sclerosis, neuromyelitis optica spectrum disorder, recurrent tumefactive disease, or an isolated monophasic course (23–25). Enhancement patterns may also reflect underlying pathological processes. In a small biopsy series, open-ring or irregular rim enhancement corresponded to macrophage infiltration and angiogenesis at the inflammatory border (26). Lesion distribution, disease-associated antibody status, and subsequent clinical events therefore refine disease attribution over time. Timely tissue diagnosis remains important when lymphoma remains a realistic concern and a diagnostically adequate target is available.

At T0, interpretation is best organized around a concordant disease model. Age, tempo, lesion geography, enhancement, diffusion, CT density, antibody status, CSF findings, and systemic staging should converge into a coherent explanation of the clinical picture. A feature should be interpreted with caution when it contributed to the reference diagnosis, was measured after treatment, or was derived from a comparator dominated by glioma. Many precise imaging estimates answer “TDL versus any primary tumor”, while the clinical decision is the narrower and more consequential TDL–PCNSL distinction. T0 should establish a reasoned prior probability and preserve a map for tissue diagnosis.

Imaging at this stage has two jobs: estimate probability and preserve a map for tissue diagnosis. Baseline contrast-enhanced MRI should be preserved for multidisciplinary review, and the most representative enhancing component should be identified before treatment alters it. If severe mass effect or rapid neurological decline makes corticosteroids unavoidable, the pretreatment lesion, intended biopsy region, and reason for treatment should be documented before corticosteroids alter the lesion characteristics.

## 4 T1. After corticosteroids: what changed

Corticosteroid response has poor etiological specificity between TDL and PCNSL because both disorders can improve clinically and shrink radiologically (2, 3, 27). A 2026 systematic review and meta-analysis of 112 biopsy-proven adult TDL cases reported in the literature estimated a pooled response to intravenous methylprednisolone of 72.3% (95% CI 57.9–83.2%) (27). The contributing evidence consisted largely of case reports and small series, so the estimate does not provide an individual likelihood ratio. It demonstrates that biopsy-proven TDL may fail to respond and that response itself cannot exclude lymphoma.

The post-treatment transformation carries more information than the label “steroid-responsive”. Symptomatic improvement may largely reflect less edema and mass effect. Reduced enhancement may reflect decreased inflammatory activity, altered blood–brain barrier permeability, direct lymphoma-cell apoptosis, or several mechanisms at once. Symptoms can improve while a diagnostically useful target remains. Enhancement can also disappear without durable clinical resolution. The informative observations are the speed, magnitude, location, and durability of change; the appearance of new lesions; and recurrence of deterioration during tapering.

Corticosteroids create two related biopsy problems. The active component of the lesion can shrink or become redistributed, leaving a trajectory planned on an old scan directed at unrepresentative tissue. Treatment can also deplete neoplastic B cells and leave macrophages, reactive astrocytes, and perivascular T cells that resemble inflammatory demyelination (28, 29). The first is a targeting problem and the second an interpretive problem. Both can occur during the same procedure, producing a technically accurate biopsy that is biologically uninformative.

The post-treatment record should reconstruct these changes in a timeline. At minimum, it should include the agent, native and dexamethasone-equivalent dose, duration, last administration, clinical course, anatomically explicit change in enhancement, latest contrast-enhanced MRI, candidate biopsy site, and planned tissue studies. Meta-analytic evidence has not identified a reliable relationship between diagnostic yield and dose, duration, or taper, although exposure definitions and treatment details were heterogeneous (3). Current evidence therefore provides no basis for a universal washout interval.

Prebiopsy and postbiopsy exposure should also be separated. Before tissue acquisition, corticosteroids can change lesion visibility, site selection, and the probability of recovering viable lymphoma. After representative tissue has been secured, subsequent treatment cannot change the diagnostic information contained in that specimen, although it may change later imaging and any repeat-sampling opportunity. A single record of “steroids during admission” collapses biologically different exposures. The T1 timeline should time-stamp the baseline scan, first dose, interval scan, CSF collection, navigation scan, and tissue acquisition whenever possible.

A useful T1 description is spatial as well as temporal. Overall lesion diameter may fall while an eccentric enhancing rim, a periventricular component, or a new distant focus retains diagnostic value. The report should state which component changed, which remained, and whether the safest representative site has shifted. This converts “partial response” into an anatomical update that can guide decision-making by neuroradiology and neurosurgery. It also prevents symptomatic improvement from obscuring a persistent tissue opportunity.

## 5 T2. Is there still a useful biopsy target

At a population level, prebiopsy corticosteroids increase the chance that the first biopsy will be nondiagnostic. Tosefsky et al. synthesized 19 studies containing 1,226 immunocompetent patients with histologically confirmed PCNSL. The pooled risk ratio was 2.1 for a nondiagnostic biopsy after corticosteroid exposure and 3.0 in the stereotactic-biopsy subgroup (3). Feldheim et al. found the same direction of effect: the first procedure confirmed PCNSL in 50 of 57 exposed patients and 88 of 91 unexposed patients (30). These data support preserving untreated diagnostic tissue whenever clinical circumstances permit.

Selected modern cohorts also document high diagnostic yield after exposure. In a four-center cohort, all 143 patients retained visible enhancement before surgery; diagnosis was obtained in 68 of 70 exposed patients and 71 of 73 unexposed patients (5). Ghoche et al. reported initial stereotactic-biopsy yields of 41 of 43 after corticosteroids and 55 of 61 without exposure. Contrast MRI was routinely obtained within 24–48 hours of biopsy, and exposure was generally brief (6). Porter, Bullis, and Binnahil also reported successful diagnosis after treatment when lesions remained available for sampling (31–33). These cohorts predominantly describe patients whose target remained visible after treatment and who proceeded to biopsy. Lesions that disappeared before surgery are underrepresented. Selected principal studies are summarized in **Table 1**.

**Table 1:** Principal studies of biopsy after prebiopsy corticosteroid exposure in primary central nervous system lymphoma.

Study/design	Lesion after corticosteroids/pathway	Diagnostic result	Main lesson/limitation
Porter et al., 2008 (31). Single-center retrospective; 109 histologically confirmed PCNSLs (107 with exposure data).	Serial imaging in 87; six had marked post-treatment change. Enhancement persisted in lesions successfully rebiopsied.	Repeat biopsy: 8/68 exposed; 5/39 unexposed. Persistent enhancement was the only identified predictor of successful repeat biopsy.	Residual enhancement may preserve a repeat-biopsy opportunity; final-diagnosis selection underrepresents lesions that disappeared before sampling.
Scheichel et al., 2021 (5). Four-center retrospective; 143 immunocompetent patients.	Every lesion still visibly enhanced before surgery.	Diagnostic surgery: 68/70 exposed; 71/73 unexposed.	Yield was high when an enhancing target remained and surgery proceeded; persistent-enhancement selection limits inference about regressed lesions.
Velasco et al., 2020 (34). Twenty-seven-center retrospective; 327 immunocompetent PCNSLs; exposure data for 280.	Of 175 exposed patients, 51 stopped corticosteroids for a median 15 days; 124 were biopsied during treatment.	Additional biopsy: exposed 20/175, unexposed 4/105; withdrawal 5/51, continued treatment 15/124. Median diagnostic delay: 64 vs 40 days.	Withdrawal was associated with a longer median diagnostic delay; the additional-biopsy proportion was similar (5/51 vs 15/124). Imaging and pathology lacked central review.
Bullis et al., 2020 (32). Single-center retrospective; 54 patients with a local tissue diagnosis; mixed biopsy/resection.	All lesions enhanced at sampling; paired MRI in four exposed patients showed regression. Exposure was usually 1–2 days.	All 18 exposed patients obtained a diagnosis.	Short exposure can remain compatible with diagnosis when enhancement persists; inclusion required a successful local tissue diagnosis.
Feldheim et al., 2024 (30). Single-center retrospective; 148 ultimately confirmed PCNSLs; mixed diagnostic procedures.	Lesion state, dose, and timing details were incomplete.	First procedure confirmed PCNSL in 50/57 exposed and 88/91 unexposed patients.	Exposure was associated with more first-procedure failures; final-diagnosis selection limits population inference.
Tosefsky et al., 2024 (3). Systematic review/meta-analysis; 19 studies, 1,226 immunocompetent histologically confirmed PCNSLs.	Among 167 exposed cases with imaging, 25 regressed; 13/14 nondiagnostic cases with imaging regressed.	Pooled risk ratio for nondiagnostic biopsy 2.1 (95% CI 1.1–4.1); stereotactic subgroup 3.0 (95% CI 1.2–7.5).	This is the best average relative-risk estimate; the 13/14 figure describes regression among failures rather than failure among all regressed lesions.
Ghoche et al., 2026 (6). Single-center cohort; 104 histologically confirmed large B-cell CNS lymphomas selected from 725 stereotactic biopsies.	Mean exposure 3.27 days; contrast MRI routinely within 24–48 h of biopsy.	Initial yield: 41/43 exposed; 55/61 unexposed.	A prompt, imaging-updated pathway achieved high diagnostic yield in this selected cohort; prolonged-exposure and failure subgroups were small.
Önder et al., 2015 (35). Selected pathology series; 25 corticosteroid-pretreated PCNSL cases undergoing stereotactic biopsy; no unexposed comparator.	Lesions reached surgery, but treatment-altered tissue showed a broad morphological spectrum.	Only 48% were diagnostically straightforward; the remainder showed substantial interpretive variability.	Reaching a lesion and obtaining interpretable lymphoma tissue are different endpoints; this pathology series cannot estimate procedural yield.

CNS, central nervous system; MRI, magnetic resonance imaging; PCNSL, primary central nervous system lymphoma.

The distinction changes bedside interpretation. A cohort beginning at histological confirmation excludes suspected patients who never reached a diagnostic procedure. A cohort requiring visible preoperative enhancement excludes those whose target vanished. Both selection processes occur after corticosteroid exposure and preferentially retain cases with accessible biopsy targets. Reported yield in the operated subgroup therefore reflects both lesion state and selection into biopsy. It describes success among patients who retained a target and reached surgery, whereas the meta-analysis describes average diagnostic risk. Current imaging determines which estimate is most relevant to the patient in front of the team.

For an individual patient, targetability has three domains. Lesion selection asks whether current imaging identifies tissue likely to retain diagnostic material and represent the suspected disease process. Procedural feasibility asks whether that tissue can be reached at acceptable risk using current navigation. Sampling feasibility asks whether the approach can obtain enough spatially informative tissue for morphology, immunophenotyping, and appropriate flow or molecular studies. Persistent enhancement opens this assessment; the three domains determine whether the remaining lesion offers a useful biopsy target.

The T2 decision point should end with an operational answer for each domain. Neuroradiology identifies the current component with the highest expected biological yield and documents how it differs from baseline. Neurosurgery confirms the trajectory and the image set used for navigation. Neuropathology specifies the minimum tissue allocation needed for morphology, myelin and axonal stains, immunophenotyping, flow cytometry, and molecular testing. A useful target exists only when these plans refer to the same lesion component. This alignment is especially important after partial regression, when old coordinates and current enhancement may point to different tissue.

Specimen adequacy is a separate postbiopsy question. It depends on tissue volume, concordance with the intended lesion component, preservation and allocation, and the cellular and architectural information left after treatment. A lesion may look suitable and still yield poor tissue. Conversely, a poor specimen may reflect handling or treatment effects despite a rational target. Recording the two endpoints separately shows where the diagnostic pathway failed.

Radiographic regression changes the target and the meaning of published denominators. In the Tosefsky meta-analysis, 25 of 167 exposed patients with relevant imaging showed regression; 13 of 14 nondiagnostic cases with relevant imaging had regressed (3). The latter figure describes regression among biopsy failures. It does not estimate failure among all regressed lesions. The clinically useful inference is narrower: regression reduces confidence in an old target and makes updated imaging essential.

When reliable enhancement persists, the available evidence favors prompt biopsy using current imaging. No cohort or meta-analysis has established a washout duration that reliably restores diagnostic yield. Velasco et al. reported additional biopsy in 20 of 175 exposed patients and 4 of 105 unexposed patients (34). Among exposed patients, additional biopsy occurred in 5 of 51 after withdrawal and 15 of 124 during treatment. Withdrawal was associated with a longer median diagnostic delay (64 vs 40 days), while no reduction in additional biopsy was observed.

For a persisting target, the biopsy plan should use the latest contrast imaging. The team should select the most representative enhancing component, review access, and plan enough tissue for routine histology and locally appropriate ancillary studies. Ghoche’s 24–48-hour imaging workflow provides one operational example (6). The specimen should arrive with the corticosteroid history, before-and-after imaging change, and sampled coordinates.

If enhancement has markedly regressed or disappeared, the team needs an active retargeting plan. Porter et al. described a lesion that disappeared after corticosteroids, leading to canceled biopsy and PCNSL diagnosis when it recurred 15 months later (31). Clinically short-interval contrast imaging should be arranged with rapid multidisciplinary review and predefined triggers for renewed tissue acquisition. Re-enhancement, neurological progression, corticosteroid dependence, or a new safer representative site should prompt immediate reassessment. The interval should reflect the clinical tempo and consequences of further delay.

Retargeting is a continuation of the diagnostic procedure, not a period of therapeutic observation. The plan should name who reviews the next scan, how quickly neurosurgical access can be reactivated, and which clinical change overrides the scheduled interval. When a different compartment becomes positive before the parenchymal lesion reappears, its diagnostic value should be judged against the same requirements of representativeness, safety, and adequacy.

Post-corticosteroid tissue requires a complete differential workup. In a selected series of 25 corticosteroid-pretreated PCNSL stereotactic biopsies, only 48% were diagnostically straightforward; the remainder produced substantial morphological and interobserver difficulty (35). A diagnosis of active demyelination should be supported by a coherent pattern of myelin loss, relative axonal preservation, and appropriately distributed macrophages, lymphocytes, and astrocytic changes (11, 28, 29). Isolated macrophages or nonspecific inflammation are insufficient. Myelin- and axon-directed stains should be integrated with a lymphoma immunohistochemical workup chosen by neuropathology or hematopathology according to morphology and local practice. Ancillary testing is most informative when guided by representative tissue, morphology, and a clearly communicated differential diagnosis.

CSF markers can change probability, and single-marker results should be distinguished from composite panels. In the prospective PAMINA study, combined CSF assessment of *MYD88* L265P and elevated IL-10 (>2 pg/mL) showed high discrimination in a selected cohort with newly diagnosed or relapsed PCNSL and controls (36). PRICELUS subsequently enrolled 255 consecutive adults with suspected PCNSL across five hospitals; 50 had CNS lymphoma, including 44 primary and six secondary cases (37). *MYD88* alone had 56.0% sensitivity and 98.5% specificity. A rule-in combination of *MYD88* positivity with elevated IL-10 or CXCL13 achieved 54.0% sensitivity and 100.0% specificity. When all three markers were negative, the reported negative predictive value was 99.4% (162/163). The 99.4% negative predictive value applies to the triple-negative composite, not to a single marker. The estimate arose in a cohort with 20% lymphoma prevalence, broad control diagnoses, six secondary CNS lymphomas, platform-specific assays, and a decision tree derived in the same cohort.

Negative predictive value rises as lymphoma prevalence falls. In PRICELUS, the space-occupying-lesion subgroup was clinically closer to the problem addressed here, although the subgroup was smaller and CXCL13 was no longer independently associated in multivariable analysis. The panel is most useful as a structured probability update. Triple negativity can materially lower probability; a lone negative *MYD88* result carries much less information. Biopsy deferral should still incorporate the starting probability, target safety, assay turnaround, and consequences of diagnostic delay.

The studies of corticosteroid effects on CSF *MYD88* address different settings. Takahashi et al. retrospectively described six glucocorticoid-exposed PCNSL patients. *MYD88* L265P was detected in CSF in 1/6 and in tissue in 5/6, with substantially fewer mutant droplets than in 16 previously reported nonexposed patients (38). PRICELUS found similar *MYD88* positivity among corticosteroid users (8/14) and nonusers (20/36; *P = 0.91*), with no meaningful subgroup difference in IL-10 or CXCL13 (37). PRICELUS provides the stronger prospective estimate, but dose was available in only 16 of 37 exposed participants and lumbar-puncture timing varied widely. The smaller study included only glucocorticoid-exposed, confirmed PCNSL cases and differed from PRICELUS in sampling context and assay workflow. Post-treatment negativity in a single assay can therefore reflect analyte depletion, sampling, or platform effects.

Conventional cytology and flow cytometry have limited sensitivity in PCNSL (3, 39). Molecular and cytokine performance varies with platform, specimen processing, cohort composition, and threshold (40–43). An early droplet-digital PCR study established technical feasibility at low DNA input, but its small selected sample does not define clinical sensitivity in suspected PCNSL (43). A convincing positive molecular result can accelerate the PCNSL diagnostic pathway, especially when tissue is unsafe or unavailable. A negative single marker offers limited reassurance, while a triple-negative composite provides stronger probability revision. Shaw et al. found a polyclonal result on retrospective PCR of an initial biopsy from a case later proven to be diffuse large B-cell lymphoma; two additional assays failed because of insufficient DNA (44). Biomarkers should update probability and prioritize action. Tissue remains the decisive test when a representative target is safely accessible or a major verified inconsistency has emerged.

## 6 T3. Does pathology still fit the course

A demyelinating diagnosis is credible when the entire chain is coherent. Pretreatment clinical and radiological evidence should support inflammatory disease, sampled coordinates should match the current target, and the specimen should be adequate. Pathology should show a coherent demyelinating pattern; isolated macrophages, perivascular lymphocytes, or treatment-altered tissue are insufficient. Subsequent imaging and clinical evolution should remain compatible with the assigned disorder (12, 13, 21, 28). Follow-up refines disease classification and tests whether the tissue diagnosis continues to explain the patient’s course.

After an initial negative or nondiagnostic biopsy, the first T3 task is to audit the specimen before deciding how much diagnostic weight the result should carry. The record should confirm which target was sampled, the number and size of fragments, and allocation to fixed and fresh studies. It should also state whether the intended ancillary tests were technically successful. A negative lymphoma result carries substantial weight in representative tissue with preserved architecture and a complete workup. The same result is weak evidence in scant macrophage-rich tissue from a regressed edge. This distinction should be explicit in the multidisciplinary conclusion. Subsequent management should be guided by specimen adequacy and clinicoradiological concordance.

A major verified inconsistency should trigger reassessment. Examples include new or disproportionately avid enhancement, relentless progression, repeated corticosteroid dependence, target–tissue mismatch, or nonspecific inflammation from a strongly suspicious lesion. Patchy incomplete myelin loss, extensive apoptosis, unusually dense T-cell inflammation, and sparse or atypical lymphoid cells do not independently establish PCNSL (28, 29, 35). In context, however, they justify renewed imaging–pathology review before further corticosteroids obscure the next diagnostic opportunity.

When the diagnosis reopens, baseline and serial imaging should first establish what changed and how the sampled site relates to the evolving lesion. The original tissue should then be reexamined with the corticosteroid history, coordinates, and updated differential. Current contrast-enhanced MRI can identify the safest representative target for new tissue. Persistent enhancement predicted repeat-biopsy success in Porter’s series (31). A prior negative CSF assay or polyclonal result should not preclude repeat tissue acquisition when pathology no longer explains the course. European Hematology Association–European Society for Medical Oncology (EHA–ESMO) guidance identifies histopathological examination of stereotactic-biopsy tissue as the diagnostic standard and advises avoiding corticosteroids before biopsy when clinically feasible (4). European Association of Neuro-Oncology (EANO) guidance recommends multidisciplinary tumor-board discussion when biopsy and resection are both plausible for a unifocal resectable lesion (45).

Reassessment should remain selective and active. A minor imaging fluctuation or nonspecific symptom seldom warrants another biopsy. The trigger is a verified inconsistency large enough to threaten the assigned diagnosis, followed by a defined search for a representative target. If no safe target exists, the team should specify the next imaging time point, clinical triggers for urgent reassessment, and any alternative compartment or site—CSF, vitreous, or a systemic lesion—that might provide safer diagnostic material. Follow-up then becomes a planned evidence-recovery strategy.

## 7 Discussion

### 7.1 What does the T0–T3 framework change

The T0–T3 sequence separates four questions that are often collapsed: what establishes the relative probability of TDL or PCNSL before treatment, what biological and anatomical change follows corticosteroids, whether a useful biopsy target exists now, and whether pathology remains concordant with the later course. Exposure predicts population-level risk. Current lesion state and workflow determine individual procedural opportunity. Longitudinal concordance determines whether the resulting diagnosis remains sufficient. This explains how an average increase in nondiagnostic biopsy can coexist with high diagnostic yield in selected exposed patients.

The sequence also changes the meaning of response. Binary steroid responsiveness has little etiological value, while the measured response pattern determines what diagnostic information remains available. Persistent enhancement opens a route to tissue diagnosis that still depends on lesion selection, procedural feasibility, and sampling feasibility. Actual specimen adequacy and interpretability are postbiopsy endpoints.

Targetability brings three disciplines’ questions into one preprocedural conversation. The neurologist and neuroradiologist identify the component most likely to represent the underlying biology, the neurosurgeon assesses access, and pathology defines the tissue volume and handling required. A positive T2 judgment means that all three domains are acceptable at that moment. An unfavorable judgment identifies the failed domain and what must change before sampling becomes useful. This vocabulary makes the decision explicit without forcing unsupported numerical weights.

Finally, the sequence provides a framework for testing whether a diagnostic conclusion remains durable. A coherent inflammatory prior, representative and adequate tissue, and a compatible course justify disease-specific management. A major verified inconsistency reopens imaging–pathology review and retargeting. This selective trigger supports decisive care while preserving a route back to tissue diagnosis.

### 7.2 What are the limitations and research priorities

The supporting biopsy literature is dominated by retrospective cohorts that often include patients who ultimately had PCNSL. Patients whose enhancing targets disappeared before surgery are underrepresented. Procedures and failure definitions vary, and modern high-yield cohorts contain few failure events. Conditioning on arrival at surgery can therefore overstate post-corticosteroid yield across the full suspected population.

The variables needed to evaluate targetability are rarely standardized. Studies usually report exposure but often omit the exact corticosteroid exposure window, rationale for lesion selection, recency of navigation imaging, planned tissue volume, specimen allocation, or coordinate concordance. Definitions and reporting of specimen adequacy are also inconsistent across studies. CSF studies differ in cellular versus cell-free DNA, volume, freeze–thaw history, assay threshold, timing, and referral prevalence. The Takahashi–PRICELUS difference shows that “steroid exposure” is too coarse a variable for molecular as well as tissue diagnosis.

Advanced imaging variables, including perfusion-derived percentage signal recovery and brain 18F-FDG PET, have not been consistently evaluated in the post-corticosteroid TDL–PCNSL setting; limited demyelinating comparator data and potential corticosteroid effects on PCNSL FDG uptake leave their incremental value for post-treatment lesion selection and retargeting uncertain (18, 46, 47).

A prospective study should enroll patients when TDL and PCNSL are first considered, before selection for biopsy or surgery. It should link corticosteroid dose and timing, serial enhancement, target selection, procedural feasibility, planned sampling, actual specimen adequacy, pathology, biomarker preanalytics, final diagnosis, and longitudinal outcomes. Relevant outcomes include time to definitive diagnosis, first-procedure failure, repeated procedures, treatment delay, neurological deterioration during observation, and missed or delayed lymphoma. This design would show whether post-treatment lesion state and workflow explain heterogeneity now attributed to exposure and whether imaging-updated retargeting improves outcomes.

## 8 Conclusion

Corticosteroid response does not distinguish PCNSL from tumefactive demyelination. Once treatment has begun, current imaging should drive the next decision. Persistent enhancement supports prompt biopsy with an updated target. Marked regression calls for planned short-interval imaging to recover a representative target. CSF biomarkers can sharpen probability, with composite patterns carrying more information than a negative single marker. After biopsy, pathology must continue to explain the sampled tissue and later course. A major verified inconsistency should reopen the diagnosis and lead to repeat biopsy when a useful target is available.
